# Facile Synthesis of Multifunctional MNPs@Chitosan-Ag Nanocomposites: Investigating SERS Substrate Potential and Antibacterial Properties

**DOI:** 10.3390/nano16100608

**Published:** 2026-05-15

**Authors:** Yeliz Akpinar

**Affiliations:** Department of Chemistry, Faculty of Arts and Sciences, Kirsehir Ahi Evran University, Kirsehir 40100, Türkiye; yeliz.akpinar@ahievran.edu.tr

**Keywords:** nanocomposite, environmental monitoring, SERS

## Abstract

Nanocomposite materials combine diverse material properties to form multifunctional structures, enhancing the efficiency of conventional applications. Particularly in environmental monitoring, such as water analysis, nanocomposites significantly improve sensitivity and lower costs associated with standard analysis methods. The SERS method is gaining popularity due to its operational simplicity, on-site applicability, and rapid results delivery. This study focused on the development of a multifunctional metal-chitosan-based nanocomposite utilizing an economical, eco-friendly approach as an SERS substrate. The resulting composite exhibits considerable preconcentration capabilities and will provide low detection limits (LOD) for future SERS applications. Specifically, magnetic nanoparticles (MNPs) were electrostatically combined with chitosan-coated silver nanoparticles (Chi-Ag NPs) to synthesize the MNPs@Chi-Ag NPs nanocomposite. CoFe_2_O_4_ NPs were prepared as MNPs. The resulting nanocomposite, which demonstrated colloidal stability after optimization, was characterized using various techniques, including UV-VIS and FTIR spectroscopy, XRD, TEM, SEM, and DLS. As a SERS substrate, the MNP@Chi-Ag NPs exhibited considerable analytical enhancement factors of (1.5 ± 0.4) × 10^6^, (7.0 ± 0.3) × 10^6^, and (1.2 ± 0.5) × 10^6^ for the detection of water contaminants BCB, CV, and MP, respectively. It was demonstrated that the substrate enhances precision and exhibits preconcentration. Finally, the MNPs@Chi-Ag NP nanocomposite demonstrates remarkable antibacterial activity, with larger inhibition zones observed at higher nanocomposite concentrations, indicating a concentration-dependent effect.

## 1. Introduction

Materials science is an important field of the natural sciences today. The preparation, examination, and smart enhancement of materials at the nanoscale have led to advancements across all fields of technology. Developing technology plays an important role in identifying and addressing problems on Earth. One of the most important of these problems is environmental pollution. Nanoscience has enabled the discovery of innovative methods in combating environmental pollution, one of the most important problems of the last century. Nanomaterials, with their specific properties, large surface area, and the ability of their surfaces to react with pollutants, have begun to play a critical role in monitoring and eliminating environmental pollution [1].

Environmental pollutants stemming from human activities such as mining, urbanization, and industrialization pose significant risks to society and human health. These factors contribute to the deterioration of global water quality and aquatic ecosystems [2,3]. Major pollutants include pesticides [4], artificial fertilizers [5], synthetic dyes [6], heavy metals [7], pharmaceutical waste [8], and microplastics [9], which are often cancerogenic, mutagenic, and resistant to degradation, leading to long-term accumulation in nature and organisms [10,11,12].

The first step in handling water pollution is to develop sensitive detection methods, and the second step is to make polluted wastewater usable again. To effectively utilize both steps, a sustainable environmental monitoring system must be established. Today, spectroscopic analysis methods are widely used to monitor water pollution [13]. Today, spectroscopic analysis methods are widely used to monitor water pollution. ICP-MS, ICP-OES, AAS, and AES instruments are used for atomic pollution analysis, while HPLC, LC-MS, and GC-MS instruments are used for molecular pollution analysis [14,15,16]. Mass spectrometry enables highly precise analysis with ng/L sensitivity, and the library properties of these instruments provide qualitative analysis with high selectivity [17]. However, high operational costs, the need for operational experts, matrix problems, limited databases, pre-treatment procedures, time-consuming, and low usability for on-site analysis are among the most significant disadvantages of these methods [18]. 

A variety of environmental monitoring techniques, such as UV-Vis Spectrophotometry, FTIR Spectrophotometry, and Fluorometry, are commonly used because they are faster, cheaper, and simpler to operate than mass spectrometers, despite having lower selectivity and sensitivity. These methods are advantageous for on-site analysis with more accessible instruments [18]. Raman spectroscopy, a technique that can be included in this group, is deemed ineffective as an analytical technique since it necessitates concentrations above 0.01 M for obtaining conventional Raman spectra. Furthermore, the Raman spectrometers previously employed are costly and large, making them unsuitable for on-site analysis. Recent advancements in Raman spectroscopy have made the technique one of the most sensitive analytical instruments, with devices that are cost-effective, battery-operated, and portable [19]. In addition to instrumental development, the surface-enhanced Raman scattering (SERS) method has been developed using plasmonic nanoparticles since the 1970s. The SERS substrate is fundamental to SERS technology and has evolved from noble metals (Au, Ag, and Pt) and transition metals to include semiconductor materials. The noble metal SERS substrate has consistently been predominant due to its exceptionally high SERS enhancement factor (EF) (10^10^–10^12^), enabling ultrasensitive detection, including at the single-molecule level [20,21]. The enhancing mechanism in SERS relies on the increased electric field from localized surface plasmon resonances, generating “Hot Spots” with concentrated electric fields. Dense hot spots significantly enhance Raman signals, necessitating interparticle distances of less than 10 nm between plasmonic structures [22].

For the last 50 years, materials science has focused on developing SERS substrates that create HOT SPOTS on the surface. Despite recent efforts to develop SERS substrates using non-plasmonic materials—such as silicon-based materials [23], TiO_2_ [24], and reduced graphene oxide [25]—the use of Au and Ag nanostructures remains widespread in the preparation and development of SERS substrates. In recent years, composite formation or surface modification with different nanomaterials has also been used to prepare SERS substrates. This approach helps to prepare a more stable and efficient substrate. Pu et al. developed a SERS substrate consisting of CC/ZnO-Ag@ZIF-8, in which CC/ZnO creates an array for silver nanoparticles, while ZIF-8 contributes to increased substrate stability [26]. Li et al. obtained a stable hydrophobic SERS substrate by attaching perfluorodecanthetiol (PFDT) modified silver nanoparticles to a polystyrene surface. In their SERS measurements with this substrate, they obtained twice the signal of the modified substrate because PFDT provides a suitable surface for attaching hydrophobic analytes. [27].

In the preparation of SERS substrates, the use of magnetic nanomaterials alongside plasmonic nanoparticles is becoming quite common. MNPs are substances with dimensions on the nanometer scale, generally ranging from 1 to 100 nanometers. At this scale, distinctive magnetic characteristics arise, differentiating them from their bulk equivalents. Magnetic nanoparticles are distinguished from others by their magnetic characteristics. Due to their superparamagnetic and ferromagnetic features, they can interact with an external magnetic field [28]. Magnetic nanoparticles impart multifunctional properties, primarily magnetism, to the materials they form composites with [29]. In SERS applications, magnetic nanoparticles embedded in a composite structure with plasmonic nanoparticles enable the nanocomposite to achieve enrichment and separation capabilities without the need for instruments. The fact that nanoparticles can be prepared using various synthesis methods and that their surfaces can be functionalized with molecules makes them targeting agents. With SERS substrates containing magnetic nanoparticles, analytes can be easily isolated from the environment through magnetic separation and enrichment, resulting in a more concentrated analyte with reduced matrix effect.

Furthermore, in such substrates, magnetic nanoparticles create a stable array for plasmonic nanoparticles, ensuring more reproducible analyses. The high analyte concentration on the substrate surface resulting from magnetic separation and enrichment increases “hot spot” formation. Therefore, enhanced SERS signals are observed in magnetic-plasmonic nanocomposite substrates [30,31,32]. Berganze et al. prepared a SERS substrate by preparing Fe_3_O_4_@Au nanoparticles using the hydrothermal method. In their study, an increase in SERS signals was observed due to the magnetic separation and preconcentration capabilities provided by the magnetic nanoparticles [33].

The use of chitosan—as a polymeric matrix and reinforcing agent—in nanocomposite structures for the aforementioned applications has become widespread. Chitosan offers various advantages, including biodegradability, biocompatibility, cost-effectiveness, and availability. Natural chitosan contains various functional groups (such as hydroxyl, amine, and amide), which make it a preferred matrix for the preparation of nanometals. [34,35]. Its porous structure makes it an effective adsorbent, and chitosan enhances the stability of the plasmonic nanoparticle solution and stabilizes existing hot spots. As a result, its use in SERS applications is highly advantageous [34,36,37].

This study aims to prepare a multifunctional metal-chitosan-based nanocomposite (MNPs@Chi-Ag NPs) using a simple, cost-effective, facile, environmentally friendly, and non-toxic method, intended for use as an SERS substrate. Since the prepared substrate is widely available and environmentally friendly, it will be used as a single-use product. In this multifunctional nanocomposite, the magnetic nanoparticles (MNPs) serve as a proconcentration and sample-preparation tool, the chitosan matrix acts as a filler and adsorbent, and the silver nanoparticles (Ag NPs) create hot spots. During the formation of the nanocomposite, MNPs and Chi-Ag NPs were combined via electrostatic interactions, without the need for any complex reactions. “This nanocomposite’s performance was examined by employing cationic and neutral water contaminants (BCB, CV, and MP) as model analytes after optimization and characterization experiments, offering proof of concept for its potential as a highly effective SERS substrate. In the final stage, the antibacterial properties of the prepared nanocomposite against both Gram-positive and Gram-negative bacteria were investigated to demonstrate the synergistic effect of chitosan, silver, and MNP.

## 2. Materials and Methods

Ferric chloride hexahydrate (FeCl_3_·6H_2_O, ≥99%, ACS reagent), cobalt chloride hexahydrate (CoCl_2_·6H_2_O, 98–102%, ACS reagent), and sodium hydroxide (NaOH, ≥97%, pellets, ACS reagent) were used for magnetic nanoparticle synthesis. Chitosan (low viscosity) and silver nitrate (AgNO_3_, ≥99%, ACS reagent), Acetic acid (CH_3_COOH, glacial, ACS reagent, ≥99.7%) were used for the preparation of silver nanoparticles. Brilliant cresyl blue (BCB), crystal violet (CV, ≥90%, ACS reagent), and methyl parathion (MP, ≥98%) were also used. All chemicals were purchased from Sigma-Aldrich (St. Louis, MO, USA) unless otherwise stated. CASO broth medium, plate count agar (PCA), and Mueller–Hinton agar were obtained from Merck (Darmstadt, Germany). Sterile paper discs were supplied by Oxoid (Basingstoke, UK).

For characterization studies, surface charge and agglomeration levels were measured using a Malvern Nano ZS90 device (Malvern, UK). Rigaku Mini-Flex X-ray powder Diffractometer (XRD, Rigaku, Tokyo, Japan) source of Cu-Kα line (λ = 1.54056 Å) was used for XRD analysis. Absorbance measurements were held by using T80+ UV-VIS PG Instruments with a quartz cuvette. Alpha, Bruker Fourier Transform Spectroscopy (Bruker, Billerica, MA, USA) was used to examine the surface functionalization of the MNP@Chi-Ag Nanostructure. For sample preparation, the KBr pellet method was used. Zeta potential and size measurements were performed using the Malvern Nano ZS90 device (Malvern, UK). For morphological characterization, the Tecnai G2 F30 Electron Microscope is a 300 kV transmission electron microscope (Tecnai G2 F30, FEI Company, Hillsboro, OR, USA), and the HITACHI SU5000 Field Emission Scanning Electron Microscope (FE-SEM; HITACHI, Tokyo, Japan) was used.

All Raman and SERS measurements performed in this study were obtained using a Jobin Yvon LabRam confocal microscopy (LabRAM, Jobin Yvon, Palaiseau, France) Raman spectrometer equipped with a charge-coupled device (CCD) detector and a holographic notch filter. The spectrograph included a 1800 grooves/mm grating, and all measurements were conducted with a 100 µm entrance slit. The instrument was equipped with 632.8 nm radiation from a He–Ne laser and a total power output of 20 mW.

### 2.1. Preparation CoFe_2_O_4_ Nanoparticles (MNPs)

**Figure 1:**
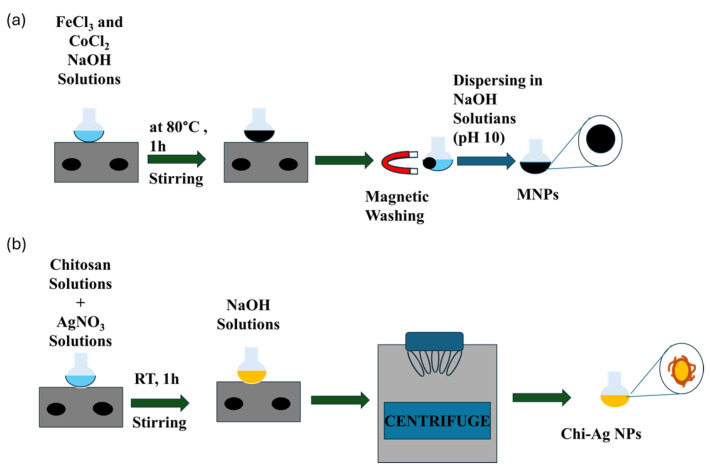
A schematic illustration of the synthesis of (**a**) MNPs and (**b**) Chi-Ag NPs.

The coprecipitation method was used to produce CoFe_2_O_4_ nanoparticles (MNPs). In a flask, a solution containing 0.0016 mol CoCl_2_ and 0.0032 mol FeCl_3_, each in 4.0 mL, was prepared. A 1.5 M NaOH solution (32.5 mL) was added at 80 °C to achieve a pH of 11, and the mixture was stirred at 600 rpm for 1 h. Using a magnet, the prepared particles were collected and washed with DI water. Finally, they were dispersed in 50.0 mL of water [38]. A schematic illustration of procedure is given at Figure 1a.

### 2.2. Preparation of Chitosan Modified Ag NPs (Chi-Ag NPs)

Chitosan-modified silver nanoparticles have been prepared by applying Mirda et al.’s study with the changing ratio of reactant [39]. Graphical scheme is given at Figure 1b. 0.025 g of chitosan was dissolved in 12.5 mL of 1% (*v*/*w*) acetic acid (CH_3_COOH) to prepare the chitosan solution, and the mixture was stirred at room temperature for 1 h at 250 rpm until fully dissolved. To prepare the Ag^+^-chitosan solution, 2.5 mL of 0.5%, 1%, 2.5%, and 5% (*w*/*v*) AgNO3 solutions were added dropwise to the previously prepared chitosan solution, which was stirred at room temperature at 200 rpm for 1 h. After that, 2.5 mL of a 5% NaOH solution was added dropwise to this solution, and after 15 min, a brown bulk precipitate solution was obtained. The brown precipitate formed indicates the formation of Ag NP-chitosan. To remove excess NaOH and chitosan, the precipitate was first washed twice with DI water and then once with a 1% acetic acid solution, centrifuged at 5000 rpm for 10 min. The supernatant was then discarded. Chi-Ag NPs precipitates were diluted in 15 mL of 0.5% acetic acid solution.

### 2.3. Preparation MNP@Chi-Ag Nanocomposites

**Figure 2:**
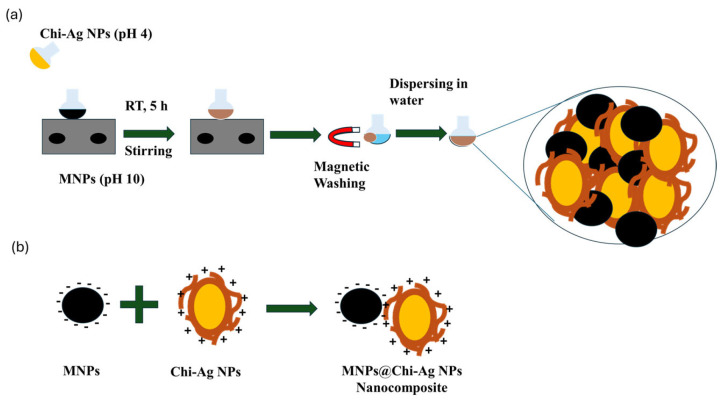
A schematic illustration of (**a**) the preparation and (**b**) its formation mechanism of the MNPs@Chi-Ag NPs nanocomposite.

2.0 mL of the prepared MNP solution was taken and diluted with 40.0 mL of DI water. The pH was adjusted to 10 using NaOH solution. 1.0 mL of the 1% AgNO_3_ colloid prepared in the previous section was added dropwise to this solution, and the mixture was stirred at room temperature for 5 h at 500 rpm. Finally, for optimization studies, the same procedure was repeated with 2.0 mL and 3.0 mL of Chi-Ag NPs colloid. After that, MNPs@Chi-Ag NPs were collected with a magnet and washed twice with DI water. Finally, they were dispersed in 15.0 mL of DI water. A schematic illustration of procedure and formation of composite are given at Figure 2a and Figure 2b, respectively.

### 2.4. Preparation of Samples for SERS Measurements

Three sets of analyses were performed. For Raman measurements, first set: 0.1 M BCB, CV in DI water, and 0.1 M Methyl Parathion in 20% (*v*/*v*) MeOH solutions are prepared, and 5 µL is dropped onto the glass surface. Secondly, 1.0 mL of MNPs-Chi nanocomposites (1 mg/mL) was added to 10.0 mL of 0.1 M solution of each of the solutions (BCB, CV, and MP) separately. To increase analyte adsorption onto the chitosan surface, the pH of the solutions was adjusted to 8; thereafter, the mixtures were shaken at 600 rpm at RT for about 30 min. The analyte adsorbed MNPs@Chi nanostructure was collected using a magnet and gently washed once with DI water. After that, the MNPs@Chi nanostructure was collected magnetically and dispersed in 20 µL DI water. 5 µL of this bulk was then dropped onto the glass surface. This procedure is applied for each analyte.

**Figure 3:**
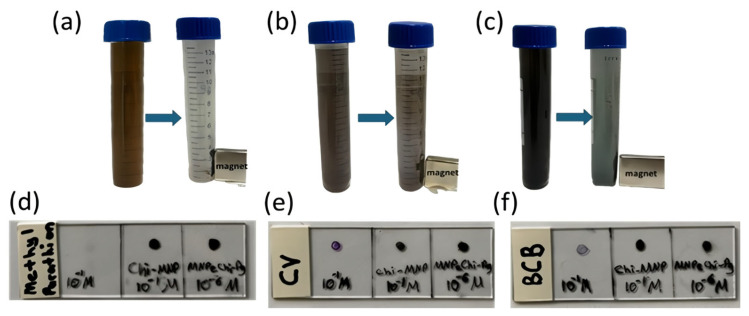
Magnetic collection and preconcentration of (**a**) MP, (**b**) CV, and (**c**) BCB analytes adsorbed onto the surface of Chi-MNPs and MNPs@Chi-Ag NPs nanocomposites. The appearance of (**d**) MP, (**e**) CV, and (**f**) BCB analytes in droplet form and in their adsorbed states on the surfaces of Chi-MNPs and MNPs@Chi-Ag NPs nanocomposites on a glass surface.

In the third set, SERS measurements were performed. 10.0 mL, 10^−6^ M, BCB, CV at DI water and 10^−6^ M Methyl Parathion at 20% (*v*/*v*) MeOH solutions were prepared. To enhance analyte adsorption onto the chitosan surface, 1.0 mL of MNPs@Chi-Ag nanocomposites (1 mg/mL) was dispersed in the analyte solutions at pH 8, separately. The same sample preparation process was applied as for the second set. After the drops dried, SERS measurements were performed. Procedure photos and sample views on the glass surface are shown in Figure 3.

### 2.5. Antibacterial Activity

The antibacterial activities of MNPs, chitosan, Chi-Ag NPs, and MNPs@Chi-Ag nanocomposite were determined using the disk diffusion method. Culture stocks (*Escherichia coli* (Gram-negative), *Staphylococcus aureus* (Gram-positive)) were prepared using CASO Broth medium. Test cultures were plated on Plate Count Agar Merck) and enumerated before experiments (10^8^ CFU/mL). PCA Medium was used for culture counting. Muller-Hinton Agar (MHA) was used in the disk diffusion method applied to determine antibacterial activity. Active cultures aged 18 h were used for inoculation. For this purpose, 100 microliters of active bacteria were inoculated onto MHA media using the spread plate method. After 5 min, sterile paper discs (Oxoid) were placed in the Petri dish at intervals sufficient for accurate measurement. Solutions of 0 mg/mL chitosan, 1 mg/mL Chi-Ag NPs, 1 mg/mL MNPs, and 1, 1.5, and 2 mg/mL MNP@Chi-Ag NPs were prepared by dispersing the particles in water, and 5 µL of each solution was dropped onto the disks, each plate set up in duplicate. The agar plates were incubated overnight at 35 ± 5 °C. Antibacterial activity was evaluated in millimeters using a millimeter scale, with the inhibition zone used as the reference [40].

## 3. Results and Discussion

### 3.1. Characterization of Chi-Ag Nanoparticles

**Figure 4:**
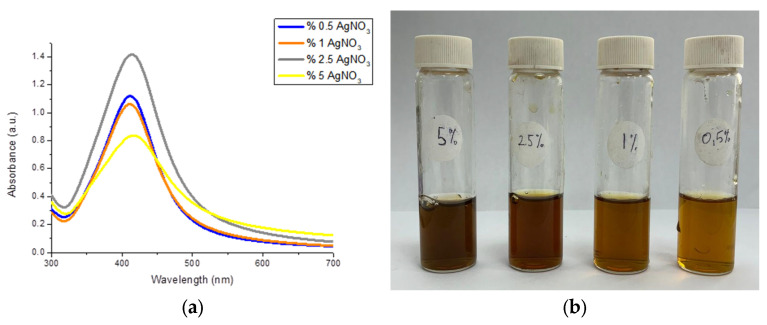
Ag-Chi nanoparticles obtained after optimization studies. (**a**) Description of what is contained in the first panel; (**b**)Absorption spectra of Ag NPs 0.5%, 1%, 2.5%, and 5% belong to (*w*/*v*) AgNO_3_ addition conditions. (**b**) Photos of Ag NPs colloids.

Chitosan-coated silver nanoparticles were synthesized efficiently and rapidly in a single step using the process developed by Mirda et al. [39]. Optimization studies were performed using AgNO_3_ solutions at various concentrations. Figure 4a displays the absorption spectra derived from UV-VIS Spectrophotometer measurements of the synthesized nanoparticles, whereas Figure 4b illustrates the visual characteristics of the created particles.

The prepared Chi-Ag NPs solutions were diluted 5-fold, and UV-VIS spectrophotometry measurements were performed. Ag NPs prepared with 5% and 2.5% AgNO_3_ absorbed at 415 nm, while Ag NPs prepared with 0.5% and 1% (*w*/*v*) AgNO_3_ absorbed at 410 nm. When the absorption peaks of the colloid were compared with the literature, Ag NPs with sizes between 10 and 30 nm were obtained in all optimization studies. However, the fact that the colloids containing 5% and 2.5% AgNO_3_ had a wider band indicates that they have a broader size distribution [41].

**Figure 5:**
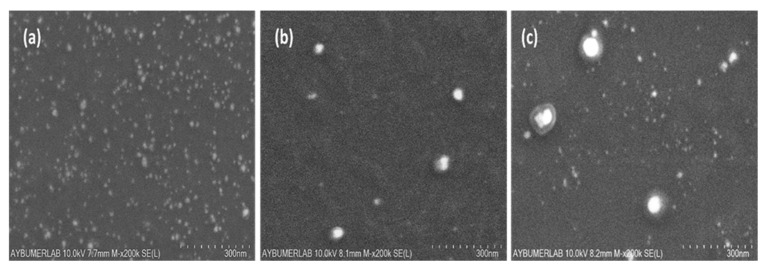
SEM images Ag NPs prepared with (**a**) A 0.5%; (**b**) 1%; (**c**); 5% (*w*/*v*) AgNO3.

SEM images of the prepared silver nanoparticles synthesized using 0.5%, 2.5%, and 5% (*w*/*v*) AgNO_3_ are shown in Figure 5a, b, and c, respectively; the image of the nanoparticles synthesized using 1% AgNO_3_ is shown in Figure 9b. The average size was calculated using ImageJ 1.54g software based on ten randomly selected nanoparticles. The sizes of silver nanoparticles prepared using 0.5%, 1%, 2.5%, and 5% (*w*/*v*) AgNO_3_ were calculated as 20.3 ± 6.4, 23.2 ± 4.5, 27.2 ± 10.3, and 35.1 ± 19.2 nm, respectively. As the amount of Ag precursor increased, the particle size increased, and a broad size distribution formed. The silver nanoparticles prepared using 0.5% (*w*/*v*) AgNO_3_, despite forming at a smaller size, exhibit a higher standard deviation compared to those prepared with 1% (*w*/*v*) AgNO_3_. The absorbance graph shown in Figure 4a and the SEM images support this conclusion. The study by Mirda et al. was used as a reference during the preparation of the Ag NPs. In their study, the Ag NPs had a size larger than 45 nm due to the high concentration of NaOH used. Since the goal of this study was to produce smaller nanoparticles, the method was modified, and the NaOH concentration (*w*/*v*) was reduced from 30% to 5%. The results obtained demonstrate that reducing the base concentration leads to a decrease in particle size [39].

**Figure 6:**
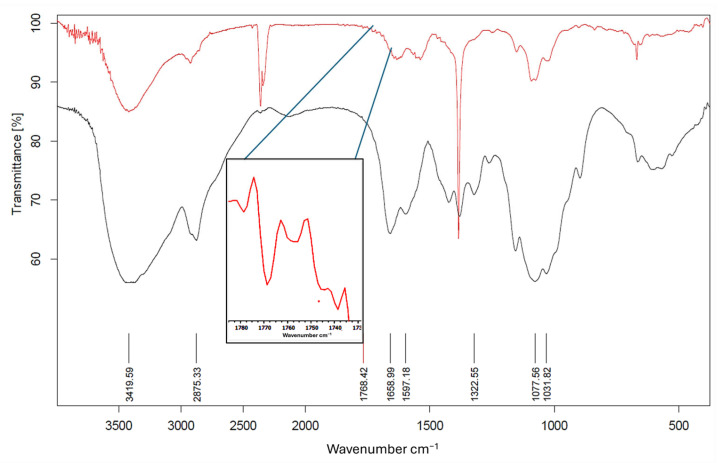
FTIR spectra of chitosan (black line) and Chi—Ag NPs (red line).

FTIR spectroscopy measurements had been performed to investigate the surface properties of Ag(0). Samples were prepared as KBr pellets. In the FTIR spectra shown in Figure 6, the black line represents pure chitosan, while the red line represents Ag NPs prepared in the presence of chitosan. The broadband at 3419 cm^−1^ in both spectra corresponds to N–H and O–H stretching, while the peak at 2875 cm^−1^ corresponds to the C–H asymmetric stretching of chitosan. Stretching of the C–O bond is represented by the bands at 1077 and 1031 cm^−1^. The bands at 1658 cm^−1^ indicate the presence of N-acetyl groups in both spectra, corresponding to the C=O stretching of amide I, and at 1322 cm^−1^, corresponding to the C–N stretching of amide III. Chi-Ag NPs samples exhibit a distinct band at 1768 cm^−1^, indicative of carbonyl stretching vibrations observed in ketones, aldehydes, and carboxylic acids. This 1768 cm^−1^ signal indicates that silver ion reduction involves the oxidation of hydroxyl groups in chitosan or its hydrolyzates [42,43]. The fact that a large portion of the chitosan-related bands is also observed in the Chi-Ag NPs air FTIR spectrum indicates that the Ag NPs have been modified with chitosan.

### 3.2. Characterization MNP@Chi-Ag Nanocomposites

**Figure 7:**
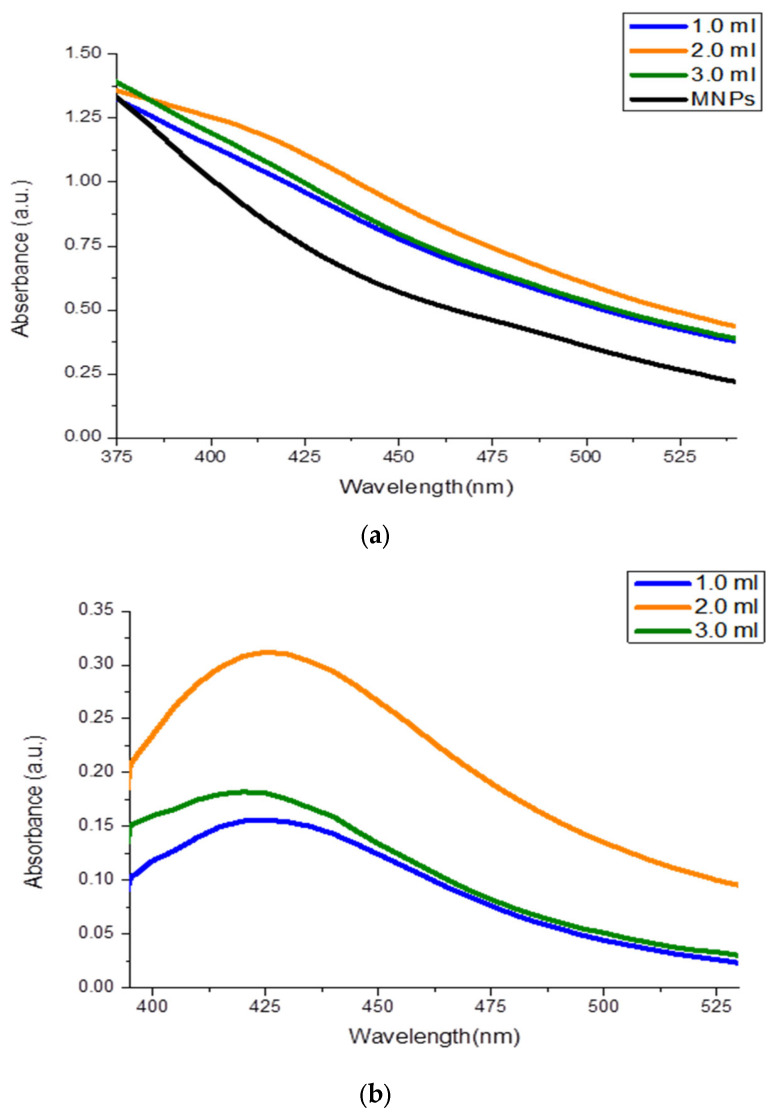
Absorption spectra of (**a**) MNPs@ Chi-Ag, which obtained 1.0 mL, 2.0 mL, and 3.0 mL Chi-Ag NPs colloid added; (**b**) MNPs@ Chi-Ag, which obtained 1.0 mL, 2.0 mL, and 3.0 mL Chi-Ag NPs colloid added via baseline correction with MNPs colloids.

MNPs@Chi-Ag NPs colloids were diluted 10 times, and their absorbances were measured using a UV-VIS Spectrophotometer. All three solutions (1.0, 2.0, and 3.0 mL additions, respectively, blue, orange, and green lines) showed absorbance around 425 nm. However, when the absorbance of the MNPs colloid at the same concentration was measured, Figure 7a shows that the MNPs in solution interfered with the signal around 375–450 nm (black line). The MNPs colloid was measured as a blank, and the same analyses were repeated. The absorbance spectrum shown in Figure 7b (orange line) indicates that the MNPs@Chi-Ag NPs, prepared by adding 2.0 mL of Chi-Ag NPs, contain more Ag NPs. When 1.0 mL of Chi-Ag NPs solution was used, the absorbance was lower than when 2.0 mL was used. This situation is a result of the surface not being saturated due to an insufficient amount of 1.0 mL Chi-Ag NP solution. Similarly, 3.0 mL of Chi-Ag NPs solution was used; the absorbance was lower than with 2.0 mL. When 3.0 mL of Chi-Ag NPs solution was used, precipitation was observed after a short time. After the negatively charged MMPs surface was filled with positively charged Chi-Ag NPs, the excess polymeric structure disrupted colloid stability and caused agglomeration. Specifically, the prepared structure lost its suspension properties because the conditions required for Chitosan dissolution were not present. This explains the lower absorbance observed compared to that of the nanocomposite colloid prepared with 2.0 mL of Chi-Ag nanoparticles.

In this study, a nanocomposite formation was designed in which positively charged chitosan-coated silver nanoparticles interacted with negatively charged MNPs via electrostatic contact. Anila et al. reported that, alongside the electrostatic interaction between chitosan and MNPs, hydrogen bonds (NH_2_–O) and chelation interactions involving amino groups and Co/Fe ions (NH_2_–Co/Fe) [44]. In here, this strong interaction of MNPs with the chitosan matrix influenced the surface plasmon resonance (SPR) of Chi-Ag NPs. Chi-AgNPs exhibited absorption within the 410 nm; in the nanocomposite, absorption shifted (redshift) to 425 nm. Interactions within the nanocomposite structure modified the local dielectric environment around the Ag NPs, resulting in an elevated refractive index and a shift of the plasmon resonance toward longer wavelengths (redshift) [45]. In addition, interactions between the functional groups of chitosan and magnetic nanoparticles (MNPs) created a non-homogeneous dielectric medium and led to changes in the interparticle distances, thereby facilitating plasmonic coupling (Ag NPs–Ag NPs) within the composite. As a result, the band broadening was observed [45,46].

**Figure 8:**
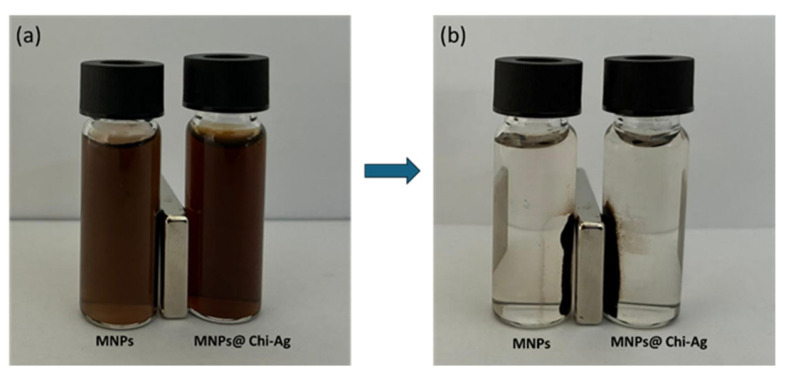
Images of the MNPs and MNPS@Chi-Ag NPs (**a**) 1 s and (**b**) 180 s after being placed next to the magnet.

A simple method was tested to determine whether the prepared composite possessed target-specific magnetic properties (i.e., the ability to collect samples from a solution using a magnet). Suspensions containing both MNPs and MNPs@Chi-Ag NPs were placed next to a magnet in separate vials. Photographs were taken at 0 s and 180 s. Both nanostructures were collected by the magnet after 180 s, and the supernatants were cleared (Figure 8a,b). Notably, the fact that the supernatant in the vial containing MNPs@Chi-Ag NPs was clear indicates that the MNPs were embedded in the chitosan matrix and that the Ag NPs were also carried toward the magnet.

**Figure 9:**
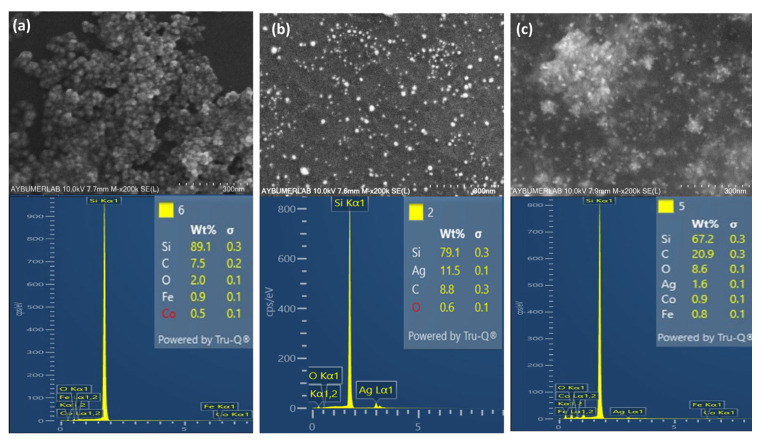
SEM images and EDX results of (**a**) MNPs; (**b**) Chi- AgNPs (which were prepared with 1% (*w*/*v*) AgNO_3_; (**c**) MNPs@Chi-Ag NPs nanocomposite.

**Figure 10:**
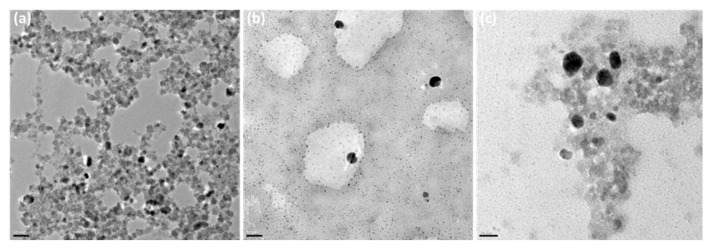
TEM images of (**a**) MNPs; (**b**) Chi-AgNPs (which were prepared with 1% (*w*/*v*) AgNO3; (**c**) MNPs@Chi-Ag NPs nanocomposite.(Scale bar is 50 nm).

Figure 9 and Figure 10 show EDX measurements, SEM, and TEM images of the nanocomposite and its components, MNPs and Chi-Ag NPs. The EDX spectra indicate a significant presence of silicon. This results from background noise generated during the production of the SEM samples, which included their deposition onto a Si wafer. In the EDX table shown in Figure 9, the elemental composition is presented in terms of weight percent (Wt%), reflecting the presence and relative distribution of elements within the nanocomposite. Due to their magnetic properties, MNPs form large agglomerates when left to stand. Figure 9a shows the morphological appearance of these agglomerates in the SEM image. EDX analysis reveals the presence of Co, Fe, and O atoms in the MNP composition. The appearance of the prepared MNPs within these agglomerates is shown in the TEM image provided in Figure 10a. From this image, the average size of the MNPs was calculated as 14.4 ± 1.8 nm using the ImageJ program after randomly selecting ten nanoparticles. As a result of the optimization studies, the SEM image and EDX spectrum of the Chi-AgNPs used to prepare the nanocomposite are shown in Figure 9b. The morphological structure appears spherical. The EDX results indicate the presence of silver within the internal structure. The TEM image of the Chi-Ag NPs shown in Figure 10b is consistent with the SEM image. The morphological structure of the MNPs@Chi-Ag NPs nanocomposite, as shown in the SEM image in Figure 9c, differs from that of the others (Figure 9a,b). The silver nanoparticles, appearing white, are seen to be surrounded by a network structure indicating the presence of chitosan, which envelops the MNPs agglomerates. In the TEM image of the same structure shown in Figure 10c, silver nanoparticles (black) and MNPs agglomerates (gray) are seen to be embedded within the chitosan bulk. In the overview TEM image of the MNPs@Chi-Ag NPs nanocomposite structure provided in Supplementary Materials Figure S1, the composite structure is more clearly visible. The TEM and SEM images demonstrate that the nanocomposite and its constituent nanostructures have been prepared as intended.

**Figure 11:**
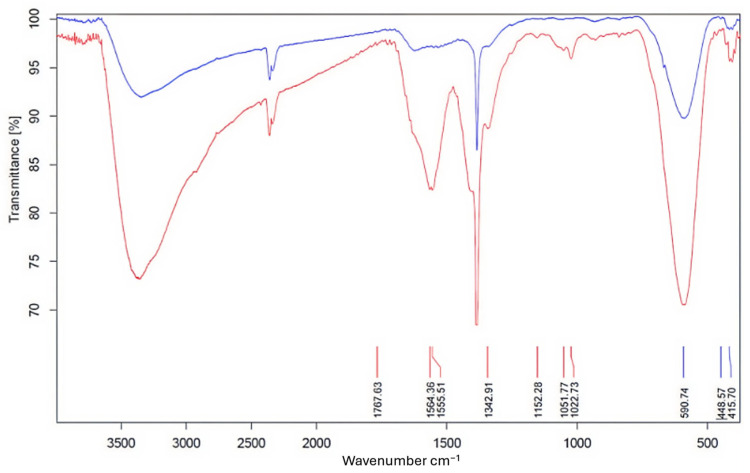
FTIR spectra of MNPs (blue line) and MNPS@Chi-Ag NPs (red line).

In Figure 11, the N–H and O–H stretching, the C–O bond stretching, and the C=O stretching of amide I and the C–N stretching of amide III (proving the presence of chiotosan) in the FTIR spectrum of MNPS@Chi-Ag NPs (given with a red line) are shown at 3396, 2920, 1051, 1564, and 1342 cm^−1^, respectively [42,43]. The wavenumbers of these bands are consistent with the FTIR spectrum of Chi-Ag NPs given in Figure 6. In addition, when the FTIR spectrum of MNPs (Figure 11, blue line) was investigated, it was found that at around 400–600 cm^−1^, two vibration bands can indicate the creation of spinel cobalt ferrite structure. There is a possible correlation between the vibration frequency of Fe^3+^−O and the strongly widened peak that was found at 590 cm^−1^. It is possible to attribute the minor peak that occurs at around 448 cm^−1^ to the vibration frequency of Co^2+^−O in the structure of ferrite [47]. Furthermore, examining the spectrum of MNPs@Chi-Ag NPs in Figure 11 shows that the presence of bands belonging to both MNPs and Chi-Ag NPs indicates that the targeted nanocomposite structure has been obtained.

The band at 1350 cm^−1^ appears in all spectra of composite and bare MNP and Chi-AgNP, indicating it is not specific to chitosan or the final composite structure. MNPs and Chi-Ag NPs were produced in an alkaline medium (NaOH) and retained alkali residues despite washing. FTIR samples were prepared by drying aqueous suspensions on KBr at 80 °C in an air ambient. The band appears to be related to carbonate/bicarbonate-like surface species, which are produced as a result of the interaction of ambient CO_2_ with hydroxylated nanoparticle surfaces and can be assigned to O–C–O asymmetric stretching vibrations of adsorbed carbonates [48,49]. In contrast, the reason the strong peak at 1350 cm^−1^ is not observed in the spectrum of pure chitosan (black line) in Figure 4 is that the sample was directly mixed with KBr in its pure solid form, and the absence of carbonate and bicarbonate species is due to the sample not being exposed to alkaline or aqueous conditions. The detected band at 2350 cm^−1^ is due to atmospheric CO_2_, which is often found in FTIR spectra [49]. Furthermore, in Figure 6, it can be seen that this band has a very low intensity in the spectrum of pure chitosan prepared directly with dry KBr (without the use of an alkaline medium or thermal treatment under ambient conditions) (black line); this confirms that the 2350 cm^−1^ band is not associated with the chitosan or composite structure but rather with the adsorption of atmospheric CO_2_ onto the surface, which is related to the FTIR sampling and/or the drying process in ambient air.

**Figure 12:**
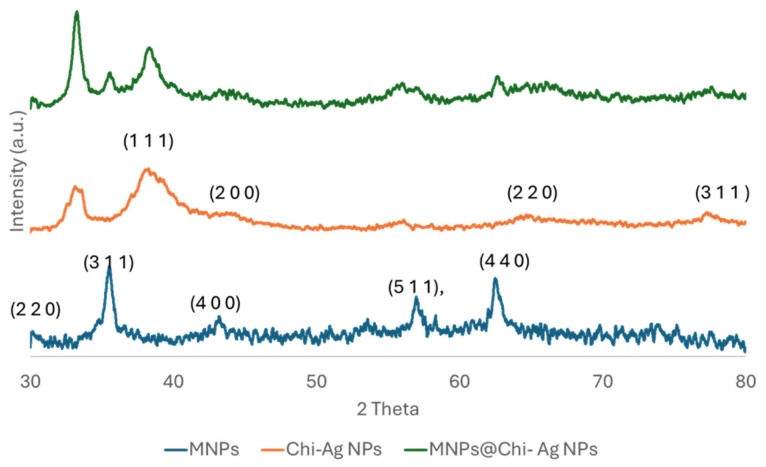
XRD patterns of MNPs (blue line), Chi Ag NPs (orange line) and MNPS@Chi-Ag NPs (green line).

To verify the crystalline structure of the nanocomposite and MNPs@Chi-Ag NPs XRD analysis of NPs was conducted using an X-ray powder diffractometer with a Cu-Kα radiation source (λ = 1.54056 Å). The JCPDS card no. 22-1086 indicates that the cobalt ferrite pattern displays five peaks between 2-theta = 30 and 2-theta = 80, specifically at 30.10, 35.47, 43.31, 56.88, and 62.49, corresponding to the (2 2 0), (3 1 1), (4 0 0), (5 1 1), and (4 4 0) (hkl) planes, respectively (Figure 12, blue line). The XRD data were analyzed by Rietveld refinement with the BGMN method in Profex. Refinement confirmed that CoFe_2_O_4_ possesses a cubic spinel structure (space group Fd-3m). The obtained lattice parameter was a = 8.4038 Å. The Rietveld Refinement yielded (R_wp_ = 3.29%, χ^2^ = 3.88). These data are considered acceptable in the literature [50,51,52,53]. The corresponding Rietveld Refinement plot is given in Supplementary Materials (Figure S2).

In the XRD pattern of Chi–Ag NPs shown in Figure 12 (orange line), the peaks at 38.74, 44.00, 64.76, and 77.21 correspond to the planes (111), (200), (220), and (311) (hkl) of the metallic silver crystal, respectively. The XRD pattern of MNPs@ChiAg NPs, shown with a green line, reveals corresponding peaks indicating the crystal structure of both cobalt ferrite and metallic silver. The XRD patterns of cobalt ferrite and metallic silver are consistent with JCPDS charts no. 22-1086 and 4-0783, respectively [53].

Size and zeta potential measurements were performed on the prepared MNPs, Chi-Ag NPs, and MNPs@Chi-Ag NPs (1.0, 2.0, and 3.0 mL) nanoparticles obtained during the optimization studies. The results of DLS Zeta Potential measurements are given in Table 1. As expected, the MNPs dispersed in a basic solution exhibited a high negative value of −33.66 ± 2.15 mV. However, since their surfaces were not coated with a very strong surfactant, the PDI and size measurements indicate that the particles exist in the form of large agglomerates due to magnetic properties, with a relatively broad size distribution [54]. The Chi-AgNPs, embedded in chitosan and suspended in a 1% acetic acid solution, exhibit a very high positive surface potential (48.57 ± 4.02) and have a 0.31 PDI and size value that can be considered monodisperse [54,55,56,57]. The nanocomposites prepared using different amounts of Chi-Ag NPs exhibit an agglomerate size that is larger than that of Chi-Ag NPs alone but smaller than that of MNPs because of the magnetic properties of MNPs.

Furthermore, the low absorbance value in the UV-VIS spectra shown in Figure 4 and the large particle size observed in the DLS measurements of the nanocomposite prepared by adding 3.0 mL of Chi-Ag NPs colloidal solution indicate that an excess of chitosan disrupts colloidal stability and causes precipitation. The surface charge of all three nanocomposites is positive, consistent with the literature. Furthermore, the PDI values of approximately 0.29 indicate a monodisperse structure.

### 3.3. SERS and Raman Measurements

This study aimed to demonstrate the proof of concept of a SERS application and to investigate the use of the prepared nanocomposite as a SERS substrate in environmental detection. Cationic dyes, BCB (Raman active) and CV (environmental pollutant), and the neutral organic molecule methyl parathion (pesticide) were selected for this aim. The literature reports the use of chitosan in the preparation of SERS substrates and the detection of negatively charged analytes via positively charged chitosan [58,59]. In this study, contrary to its common use, the chitosan surface was used as a SERS substrate for the detection of positively and neutrally charged analytes. The pZc value of chitosan is reported in the literature as 7.6. It has been reported that the surface becomes negatively charged above this value [60]. In another study, Ramanery et al. reported that the pKa of chitosan is approximately 6.5; at pH values greater than 6.5, protonation decreases, the NH_3_^+^ groups on the surface convert to NH_2_, electrostatic repulsion diminishes, and hydrogen bonds and hydrophobic interactions begin to form [61]. Alorabi reported that when the pH of the chitosan surface was raised above 7, cationic dyes such as Machite Green and CV were adsorbed onto the chitosan surface with higher efficiency [62]. Vafakish et al. analyzed the cationic Methylene Blue dye using a chitosan-based SERS substrate they prepared [35]. In this study, the chitosan surface was charged negatively or neutrally by adjusting the pH to 8, allowing cationic analytes to adhere to the surface. Furthermore, adjusting the pH to 8 eliminated the strong positive surface charge of chitosan; as its colloidal stability was disrupted during analyte adsorption, the nanocomposite tended to agglomerate and was collected more rapidly by the magnet, along with the analytes present on its surface.

Three different trials were conducted for each analyte in the SERS application Figure 3a–f. (1.) 10^−1^ M analyte was directly dropped onto the glass surface. (2.) 10^−1^ M analyte and Chi-MNPs nanoparticles were agitated together and collected using a magnet. The paste-like sample was dropped onto the substrate. (3.) The analytes were agitated with 10^−6^ M analytes and MNPs@Chi-Ag NPsnanocomposites, and the resulting paste was dropped onto the glass surface using a magnet. Before the start of Raman and SERS measurements, a blank measurement was conducted by taking the SERS spectrum of MNPs@Chi-Ag NPsnanocomposites, which is given in the Supplementary Materials (Figure S3).

Analytical Enhancement factor (AE) was calculated for three analytes. The Equation for AE calculation is given below:

$$AEF=\frac{I_{SERS}/C_{SERS}}{I_{Raman}/C_{Raman}}$$

*I_SERS_* and *I_Raman_* denote the intensities of the average SERS and conventional Raman signals, respectively, whereas *C_Raman_* and *C_SERS_* represent the analyte concentrations in the Raman and SERS measurements, respectively [63].

**Figure 13:**
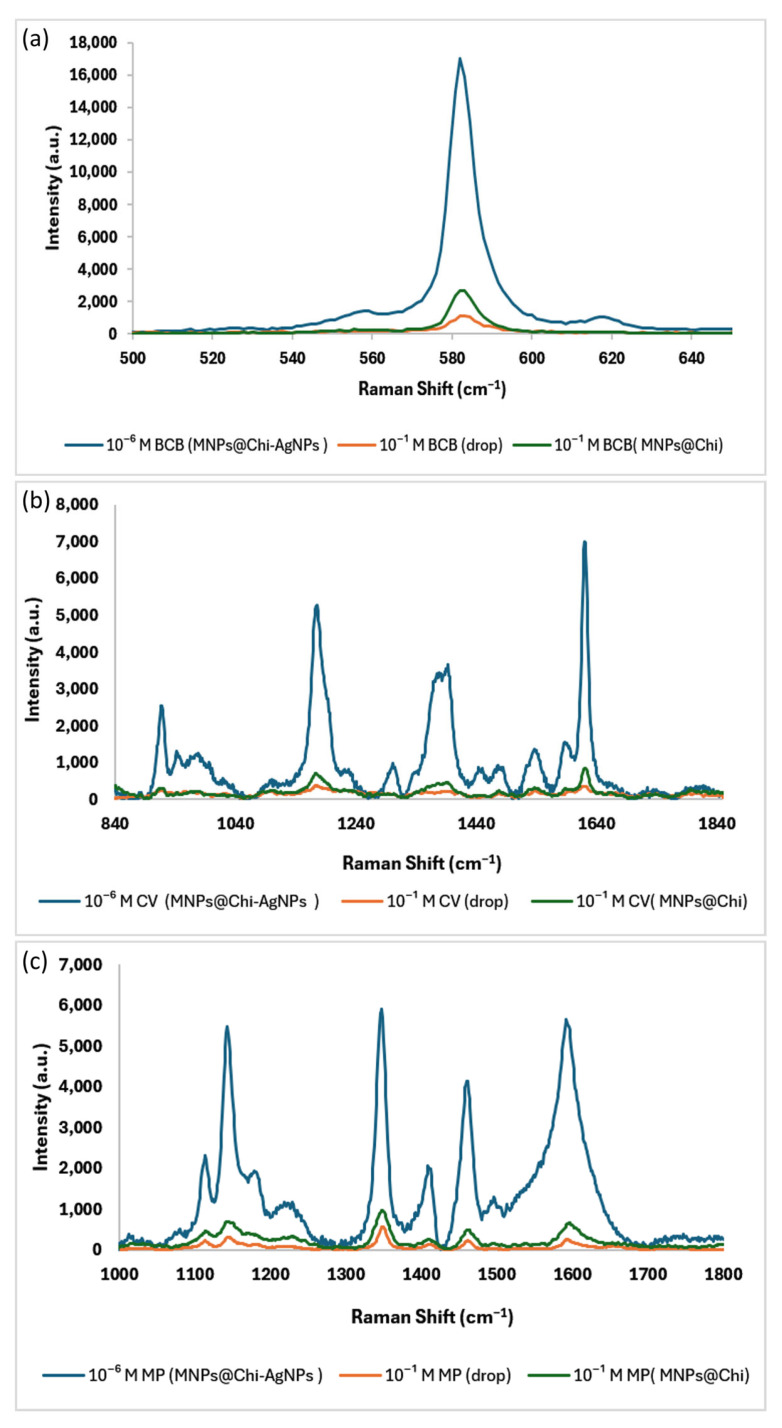
SERS and Raman spectra of (**a**) BCB, (**b**) CV, and (**c**) Methyl Parathion.

The SERS and Raman spectra of BCB shown in Figure 13a reveal the characteristic Raman shift of BCB at 581 cm^−1^ [64]. Samples were analyzed by dropping 10^−1^ M BCB and using a 10^−6^ M BCB MNPs@Chi-Ag NPs SERS substrate, and the analytical enhancement factor (AEF) [63] was calculated as (1.5 ± 0.4) × 10^6^ (*n* = 3). The SERS and Raman spectra of CV, as depicted in Figure 13b, exhibit four prominent peaks. The prominent peak at around 1620 cm^−1^ is ascribed to in-plane C-C vibrations of stretching of the ring. A distinct peak at 1397 cm^−1^ probably arises from the superposition of in-plane C-C stretching and N-phenyl in-plane stretching vibrations. Furthermore, the spectra exhibit peaks at 1172 cm^−1^ and 919 cm^−1^, corresponding to in-plane deformation vibrations of the benzene ring and skeletal vibrations of radical orientation [65]. Using the concentrations in the BCB and the main peak intensity at 1620 cm^−1^, the AEF value for CV detection was calculated as (7.0 ± 0.3) × 10^6^ (*n* = 3). Finally, the SERS and Raman spectra of the MP are shown in Figure 13c. The most significant Raman band in methyl parathion is observed at 1345 cm^−1^, corresponding to the symmetrical stretching vibration of the nitro group. The bands at 1144 cm^−1^ correspond to C–N stretching and symmetrical ring stretching, and the peak at 1593 cm^−1^ is attributable to the C=C stretching of the phenyl ring [66,67]. AEF value of MP detection was calculated as (1.2 ± 0.5) × 10^6^, (*n* = 3). The literature reports that AEF values ranging from 10^6^ to 10^8^ are considered acceptable [68,69].

**Figure 14:**
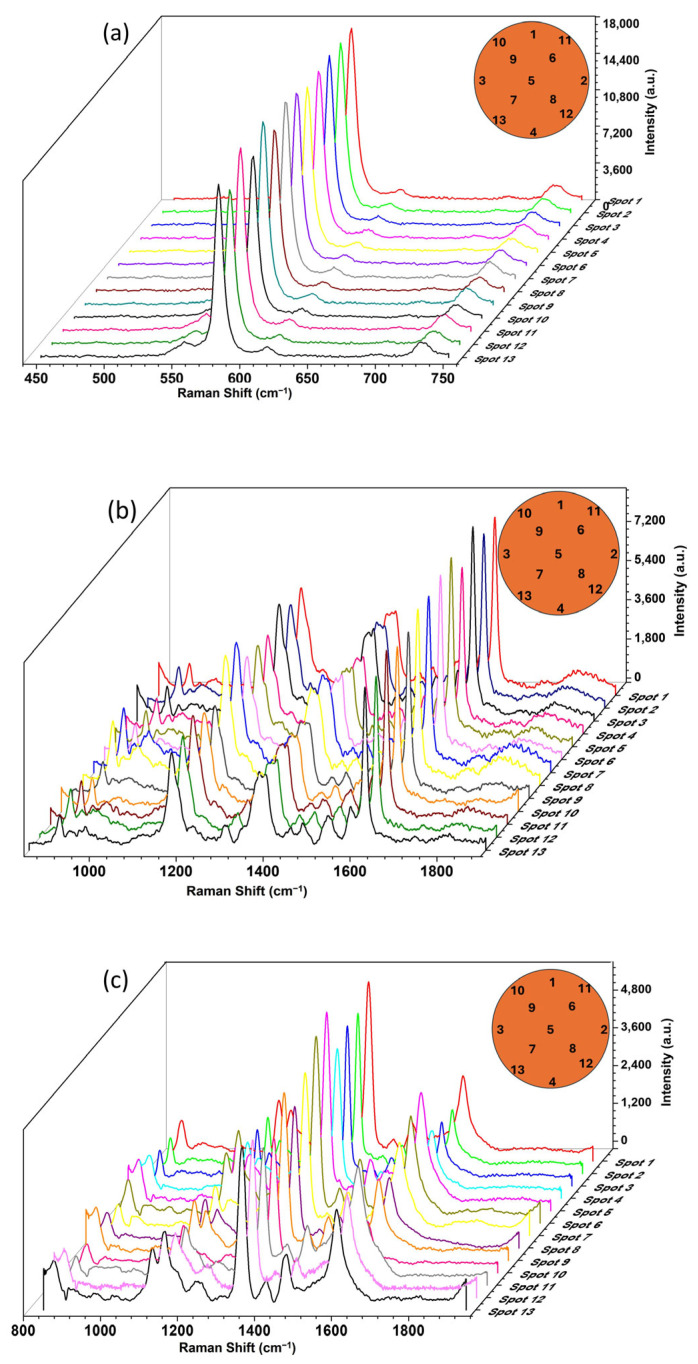
SERS spectra measured of 10^−6^ M (**a**) BCB, (**b**) CV, and (**c**) MP randomly selected thirteen different spots on the MNPs@Chi-Ag NPs nanocomposite drops as a SERS substrate. The locations of the measurement spots are shown as insets on the graphs.

The spot-to-spot reproducibility was verified by collecting SERS spectra at 13 random positions on the MNPs@Chi-Ag NPs substrate surface for each analyte at 10^−6^ M. SERS spectra and spot locations on the sample are given in Figure 14. %RSD was calculated as 4.84 for BCB band at 581 cm^−1^, 7.31 for CV band at 1620 cm^−1^, and 12.21 for MP band at 1345 cm^−1^ (*n* = 13). Low %RSD values (<20) indicate that the hotspots created by Ag nanoparticles within the substrate are distributed homogeneously across the substrate, demonstrating acceptable reproducibility. The relatively high RSD value for MP compared to the other two analytes is attributed to the lower water solubility of this substance and its relatively weaker interaction with the composite surface.

**Figure 15:**
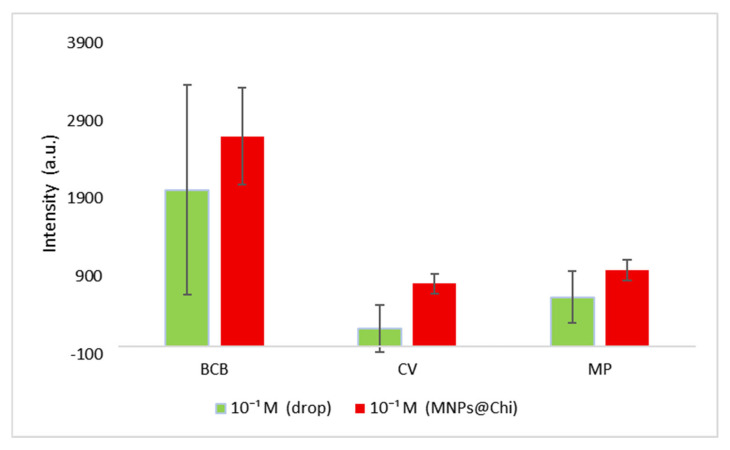
Comparison of 10^−1^ M BCB, CV, and MP Raman measurements performed using the drop and MNPS@Chi method.

Figure 15 shows a comparison of SERS measurements for all three analytes at a concentration of 10^−1^ M, both when directly dropped onto the glass surface and when prepared using the MNPs-Chi nanomaterial as described in Section 2.4, “Preparation of Samples for SERS Measurements.” This measurement demonstrated that chitosan retains analytes on its surface at pH 8, thereby enabling preconcentration. For all three analytes, when MNPs-Chi was used as an adsorbent, higher intensities were obtained than in Raman measurements from droplets at the same concentration. This indicates that the analyte molecules are densely trapped within the MNPs-Chi pellet collected using the magnet. Chitosan, owing to its amino (–NH_2_) and hydroxyl (–OH) functional groups, might adhere pesticides via electrostatic interactions, van der Waals forces, hydrogen bonding, and hydrophobic interactions [70]. On the other hand, when examining the substrates prepared for SERS and Raman measurements shown in Figure 3d–f, a “coffee ring” formation was observed in the droplet.

In contrast, the MNPs-Chi and MNPs@Chi-Ag NPs nanocomposites, which were collected together with the analyte and dispensed, exhibited a more homogeneous appearance. During the analysis, measurements were taken at five points on the sample circles on the glass surface, and the results are shown in the graph in Figure 15. Compared with MNPs-Chi, significantly wider error bars were observed in the droplet-prepared samples for all three analytes. This is due to the inherent heterogeneity arising from “coffee ring” formation in the droplet-prepared samples. Therefore, this demonstrates that chitosan-based substrates yield higher sensitivity and precision in both Raman and SERS measurements compared to direct droplet measurements.

### 3.4. Antibacterial Activity

**Figure 16:**
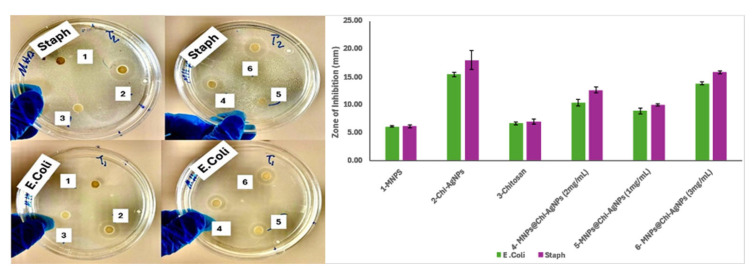
Antibacterial activities of 1–MNPS, 2–Chi-AgNPs, 3–Chitosan, 4–MNPs@Chi-AgNPs (2 mg/mL), 5–MNPs@Chi-AgNPs (1 mg/mL), 6–MNPs@Chi-AgNPs (3 mg/mL).

Figure 16 shows the antibacterial activity of 1 mg/ mL MNPS, Chi-Ag NPs, and Chitosan. Various dosages of MNPs@Chi-Ag NPs (2, 1, 3 mg/mL) were determined by the disk diffusion method, and the tests were conducted using *Escherichia coli* (*E. coli*) and *Staphylococcus aureus* (*Staph*). Upon examining the zone diameters in Figure 16, the antibacterial activity of MNPs is negligible in both cultures. On the contrary, when the antibacterial effects of Chi-Ag NPs, chitosan, and nanocomposites were examined, zone diameters at an acceptable level were observed [71]. The study found that the Gram reactions of the test bacteria varied significantly depending on the material used. When both Gram-positive (*Staphylococcus aureus*) and Gram-negative (*E. coli*) strains are tested for each sample, it is observed that Staphylococcus aureus exhibits larger inhibition zones. The reason for this observation is that gram-positive bacteria have a strong peptidoglycan layer and lack an outer lipopolysaccharide membrane. This structure facilitates the adherence of nanoparticles and the infiltration of Ag^+^ ions. Conversely, the outer membrane of *E. coli* functions as a permeability barrier, restricting the passage of Ag^+^ and the attachment of nanoparticles [71,72]. The antibacterial mechanism of chitosan-silver nanoparticles (Chi–AgNPs) likely entails multiple synergistic effects: the liberation of Ag^+^ ions that impair bacterial enzymatic and respiratory functions, the production of reactive oxygen species (ROS) resulting in oxidative damage, and electrostatic interactions between the positively charged chitosan and negatively charged bacterial surfaces that enhance nanoparticle adhesion and membrane permeability. The chitosan matrix also improves the stability of silver nanoparticles (AgNPs), facilitating sustained ion release and extended antibacterial efficacy [71,72,73]. The lower antibacterial activity of MNP suggests that the antibacterial effect of the nanocomposite is mainly due to the silver component. Literature studies show that the addition of silver nanoparticles to similarly prepared cobalt ferrite systems gives a similar increase in antibacterial activity [74,75]. Riyatun et al. reported similar antibacterial mechanisms for Ag NPs decorated cobalt ferrite nanocomposites, which involve the release of Ag^+^ ions, modification of the membrane permeability, and activation of oxidative stress [76]. In their study, Xu et al. prepared an Ag/AgCl/CoFe_2_O_4_ nanocomposite and found that the antibacterial effect was attributed to the silver species, while they were unable to detect antibacterial activity in the cobalt ferrite nanoparticles [72,77]. As expected, the prepared nanocomposites have shown a stronger antibacterial effect than either MNPs or chitosan alone. Furthermore, the fact that the prepared nanocomposite produces a wider Inhibition zone when used at increasing doses is consistent with concentration experiments conducted in the literature for the same purpose [71].

## 4. Conclusions

In this study, a multifunctional nanocomposite with magnetic, optical, and surface-active properties was prepared. The potential of this nanocomposite, prepared using a simple, rapid, non-toxic, and eco-friendly method, to be used as a disposable SERS substrate was investigated. The morphology, optical features, crystalline structure, and surface functional structure of the MNPs@Chitosan-Ag NPs nanocomposite material prepared using this method have been demonstrated and verified through characterization studies. In particular, when chitosan was used as an adsorbent, unlike conventional analyses of anionic species, the prepared SERS substrate successfully demonstrated proof-of-concept detection of cationic and hydrophobic neutral water pollutants. An acceptable analytical enhancement factor of 10^6^ was obtained for each of the three analytes. Furthermore, when used solely as an adsorbent in standard Raman measurements, the achievement of lower RSD values compared to droplet-based measurements demonstrates the substrate’s repeatability and higher signal homogeneity. Thanks to the magnetic nanoparticles embedded within the chitosan matrix, the Ag NPs have come closer together, leading to increased hot spot formation. Additionally, the applied procedure has demonstrated an effective preconcentration function, significantly enhancing the Raman signal of the nanocomposite substrate by using a single magnet to collect analyte molecules into a small area. Raman spectroscopy measurements have demonstrated that the prepared multifunctional MNPs@Chi-Ag NPs nanocomposite can serve not only as a SERS substrate but also as an adsorbent capable of adsorbing molecules from solution. Therefore, it is anticipated that in the future, positively charged molecules will be able to accumulate on the nanocomposite surface more efficiently due to strong electrostatic interactions. Since the use of an effective adsorbent is intended in future studies, particularly in water purification applications, its antibacterial properties have been tested, and effective results have been obtained. Antibacterial activity results demonstrated that MNPs@Chi–AgNP nanocomposites had concentration-dependent antibacterial activity and the inhibition zones were increased with increasing concentration. Chi–AgNPs exhibited the largest inhibition zones, but the nanocomposite showed significant antibacterial activity compared with MNPs and chitosan alone.

In the future, this nanocomposite will be used as a SERS substrate to develop a quantitative analytical method, and analyses of real-world samples (tap water, lake, or river) will be conducted.

## Figures and Tables

**Table 1:** Results of DLS measurements.

Nonostructure	PDI	Size (d, nm)	Zeta Potential (mV)
MNPs	0.45	504.30 ± 64.63	−33.66 ± 2.15
Chi-Ag NPs	0.31	21.72 ± 9.99	48.57 ± 4.02
MNPs@Chi-AgNPs (1.0) mL)	0.23	311.17 ± 191.61	36.56 ± 1.33
MNPs@Chi-AgNPs (2.0) mL)	0.29	174.43 ± 39.64	41.06 ± 1.77
MNPs@Chi-AgNPs (3.0) mL)	0.26	362.90 ± 22.73	40.77 ± 0.85

## Data Availability

The data presented in this study are available within the article and Supplementary Materials.

## Supplementary Materials

The following supporting information can be downloaded at: https://www.mdpi.com/article/10.3390/nano16100608/s1, Figure S1. Overview TEM image of MNPs@Chi-Ag NPs nanocomposite. Figure S2. Rietveld refinement of XRD pattern of CoFe_2_O_4_ NPs was performed using BGMN method in Profex. Figure S3. SERS spectrum of Chi-Ag NPs.

## Abbreviations

The following abbreviations are used in this manuscript:

AEF	Analytical Enhancement Factor
Ag NPs	Silver Nanoparticles
BCB	Brillant Creysal Blue
Chi	Chitosan
CV	Crystal Violet
*E. Coli*	Escherichia coli
FTIR	Fourier Transform Infrared
MNPS	Magnetic Nanoparticle
MP	Methyl Parathion
SEM	Scanning Electron Microscope
SERS	Surface Enhancement Raman Scattering
Staph	Staphylococcus aureus
TEM	Transmission Electron Microscopy
UV-Vis	Ultraviolet-Visible
XRD	X-Ray Diffraction
