# Supplementary material for: Facile Synthesis of Multifunctional MNPs@Chitosan-Ag Nanocomposites: Investigating SERS Substrate Potential and Antibacterial Properties
Source: Nanomaterials (Basel). 2026 May 15;16(10):608. doi: 10.3390/nano16100608 (PMC13210128; doi:10.3390/nano16100608)
Supplement: Supplementary file 1 [file nanomaterials-16-00608-s001.zip › nanomaterials-4290504-supplementary.pdf]

nanomaterials

Article

Facile Synthesis of Multifunctional MNPs@Chitosan-Ag Nano-composites: Investigating SERS Substrate Potential and Anti-bacterial Properties

Yeliz Akpinar

Department of Chemistry, Faculty of Arts and Sciences, Kirsehir Ahi Evran University, Kirsehir, 40100, Tü-rkiye;
yeliz.akpinar@ahievran.edu.tr

Results

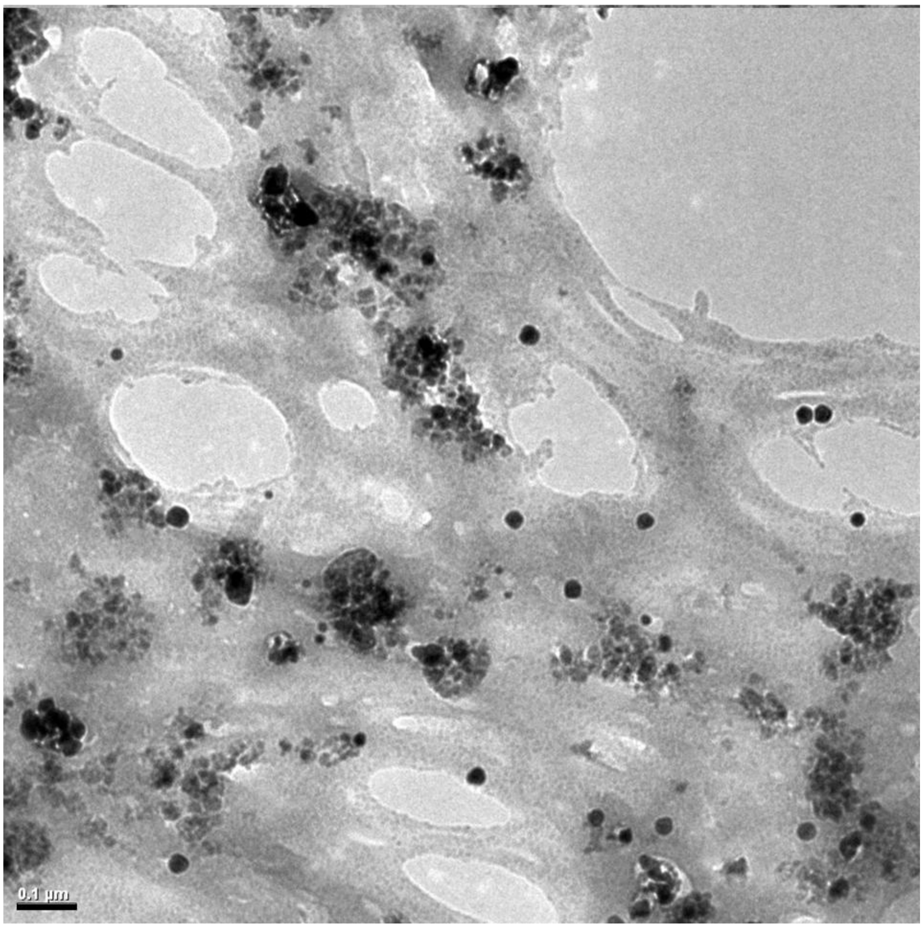

Figure S1. Overview TEM image of MNPs@Chi-Ag NPs nanocomposite

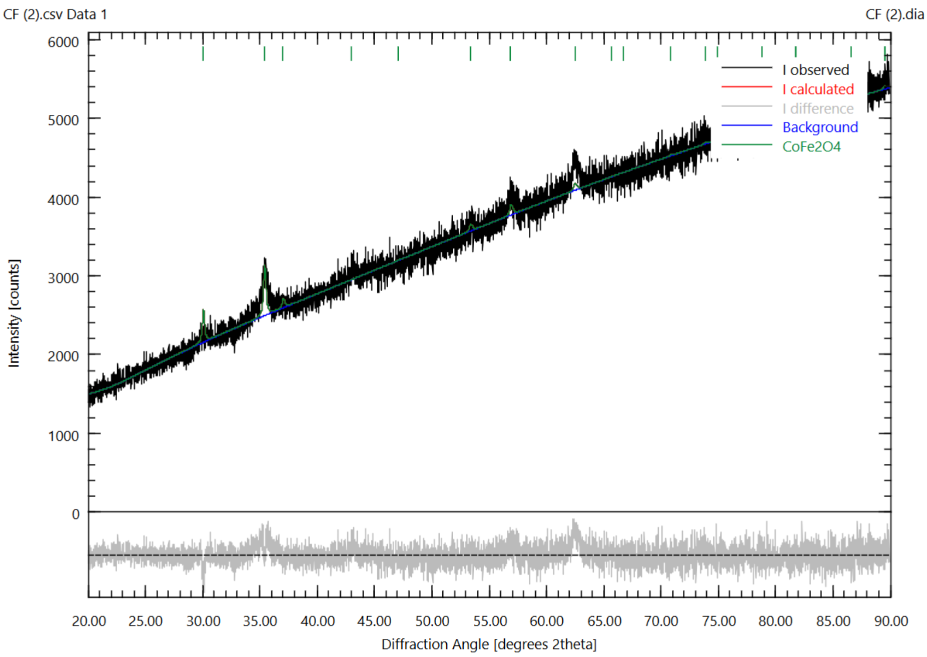

Figure S2. Rietveld refinement of XRD pattern of $CoFe_2O_4$ NPs was performed using BGMN method in Profex.

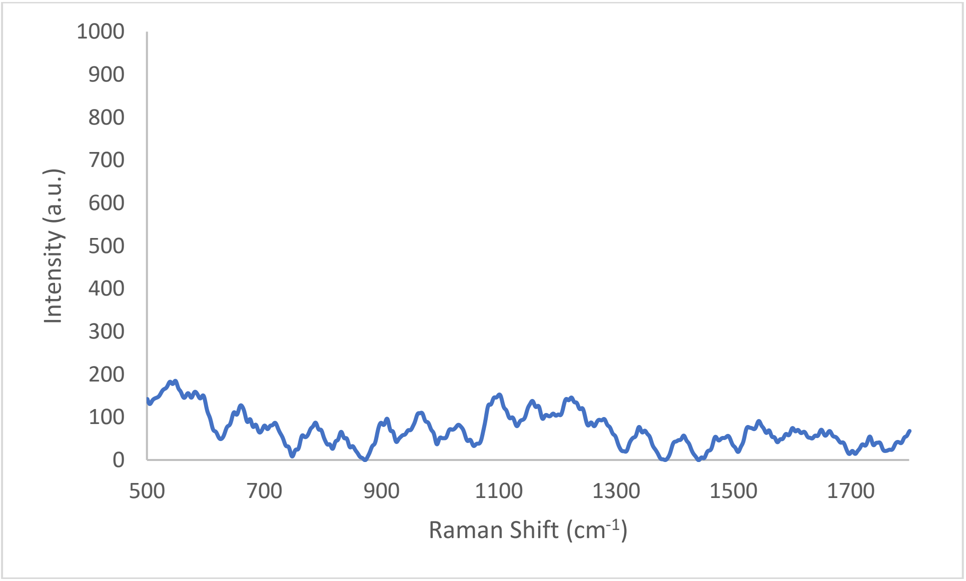

Figure S3. SERS spectrum of Chi-Ag NPs

https://doi.org/10.3390/nano16100608
